# Increase in glycemic set point, alongside a decrease in waist circumference, in the non-diabetic population during the Japanese National Intervention Program for metabolic syndrome: A single-center, large-scale, matched-pair analysis

**DOI:** 10.1371/journal.pone.0268450

**Published:** 2022-08-10

**Authors:** Takuya Sugiyama, Yuya Yamada, Yoshito Ito, Ryohei Mineo, Ryuya Iwamoto, Sachiko Tamba, Takashi Fujimoto, Koji Yamamoto, Yuji Matsuzawa

**Affiliations:** 1 Department of Endocrinology and Metabolism, Sumitomo Hospital, Osaka, Japan; 2 Physical Check-up Center, Sumitomo Hospital, Osaka, Japan; Università degli Studi di Milano, ITALY

## Abstract

**Background:**

In 2008, the Japanese government implemented a National Intervention Program for metabolic syndrome. Low-risk individuals were not direct targets of this intervention. Nevertheless, they were indirectly enlightened by this massive campaign. Documentation of the metabolic shifts in low-risk individuals following the program launch may inform public health policy regarding approaches to metabolic risks in the general population.

**Methods:**

We conducted a cross-sectional analysis of data from non-diabetic participants who underwent general health check-ups at the Physical Check-up Center of Sumitomo Hospital. Participants during 2007–2008 were pair-matched with those during 2015–2016 with respect to sex, age, smoking status, hemoglobin level, and red blood cell (RBC) count. Each participant was included only once in the study.

**Results:**

Totals of 3,140 men and 2,048 women were pair-matched. The non-diabetic participants showed lower waist circumference, blood pressure, heart rate, and serum lipid concentrations during the second study period. In contrast, the entire distributions of fasting plasma glucose (FPG) concentration in both sexes and glycated hemoglobin (HbA_1c_) in women were shifted upwards. In men, Δ FPG was +1.6 mg/dL (*P* < 0.001) and Δ HbA_1c_ was ±0% (*P* = 0.6). In women, Δ FPG was +3.0 mg/dL (*P* < 0.001), and Δ HbA_1c_ was +0.1% (*P* < 0.001). Δ Homeostasis model assessment of β-cell function was −6.6 in men (P < 0.001) and −10.3 in women (*P* < 0.001). The homeostasis model assessment of insulin resistance did not change significantly.

**Conclusions:**

The “glycemic set point” has increased in non-diabetic people in Japan during recent years. Lifestyle or environmental changes may have caused this metabolic shift through obesity-independent pathways, possibly through effects on pancreatic β-cell function. The underlying mechanism awaits further investigation.

## Introduction

Specific interactions between genetic risk factors and environmental risk factors are the hallmark of type 2 diabetes (T2D). Epidemiological evidence indicates that the pervasive transformation of the social environment worldwide underlies the threat posed by the T2D pandemic to global public health [1]. In East Asian people, a relatively small change in the social environment and slight increases in obesity and insulin resistance are sufficient for the development of T2D, because the genetically determined function of their pancreatic β-cells is significantly lower than those of other ethnic groups [2].

The Japanese government implemented a “lifestyle intervention” component within the national healthcare system in 2008. This aimed to systematically reduce the prevalence and severity of metabolic diseases, including T2D [3]. All health insurers were obliged to provide high-risk individuals with professional counseling to facilitate lifestyle remediation, but low-risk individuals were not counseled. Nevertheless, they were likely to have been influenced by this massive campaign regarding the disease risks associated with modern lifestyles. The impact of the campaign was sufficient in the general population for the term “metabolic syndrome” to enter the mainstream Japanese language. However, it is unknown whether low-risk individuals have experienced changes in metabolism following the implementation of the program.

Here, we aimed to determine whether the non-diabetic population underwent significant changes in metabolic parameters after the implementation of the Metabolic Syndrome Intervention Program. To this end, we conducted a large-scale, matched-pair, retrospective population analysis of data from non-diabetic participants who underwent general health check-ups at the Physical Check-up Center of Sumitomo Hospital (Osaka, Japan).

## Materials and methods

### Participants

**Fig 1:**
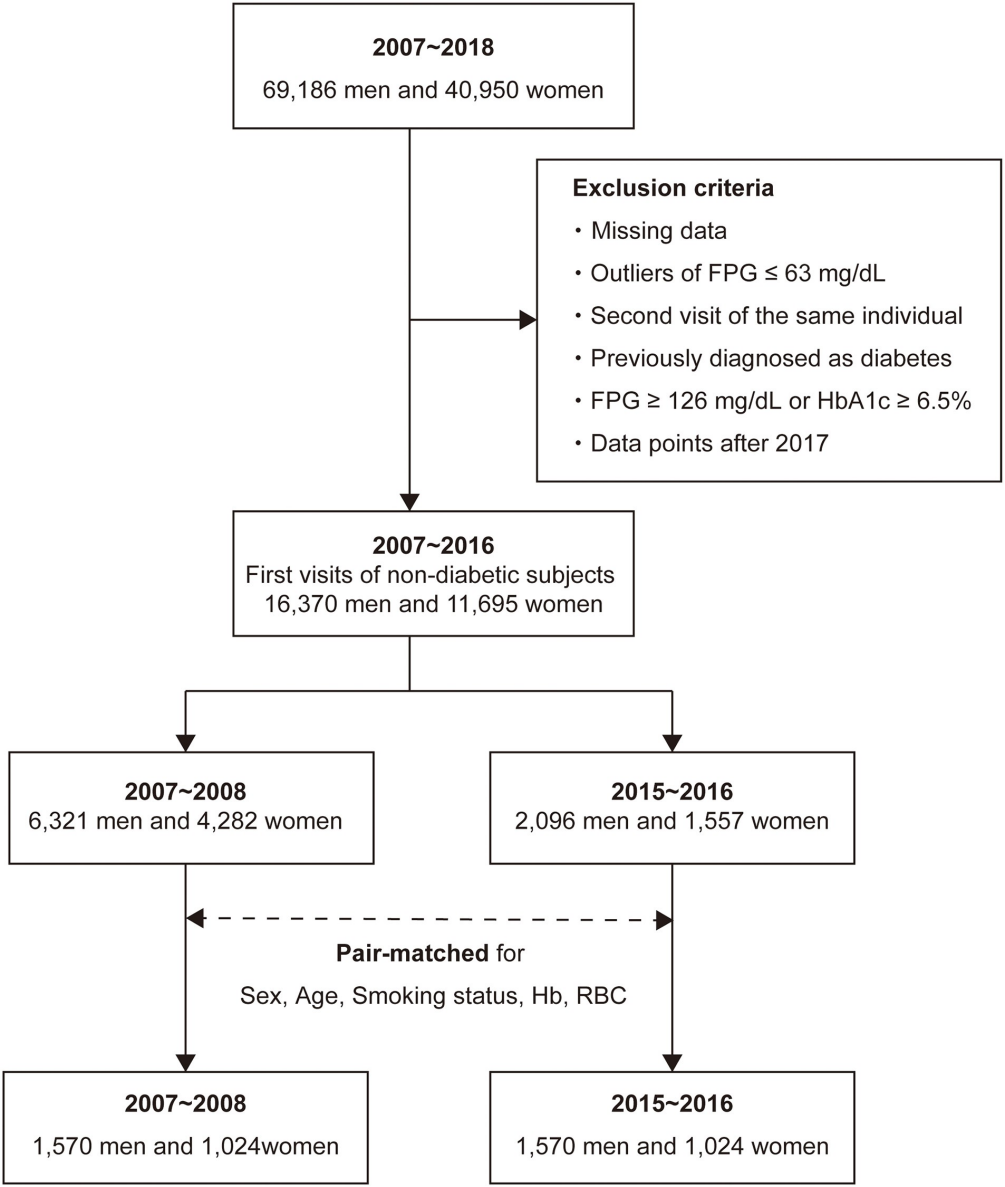
Flowchart of the study design.

First, we retrospectively interrogated 110,136 data points, representing 69,186 men and 40,950 women, in the health check-up data collected at the Physical Check-up Center between April 2007 and June 2018 (Fig 1). Health check-up programs for Japanese citizens are designed to detect diseases and their risk factors at an early stage. Often, individuals undergo such check-ups spontaneously, most are health-conscious, and none have severe conditions. It is not rare for the same individual to undergo more than one health check-up.

The exclusion criteria were as follows: (i) missing data (for 17,801 data points, 16% of the total); (ii) outlier fasting plasma glucose (FPG) concentrations ≤ 63 mg/dL, because they are not suitable for the calculation of homeostasis model assessment of β-cell function (HOMA-β) (for 41 data points); (iii) data for individuals collected at their second or later visit to our center; (iv) a previous diagnosis of diabetes mellitus (945 men and 174 women among the 19,552 men and 13,036 women remaining after the application of criteria (i) to (iii); (v) likelihood of having diabetes mellitus: FPG ≥ 126 mg/dL or glycated hemoglobin (HbA_1c_) ≥ 6.5% (981 men and 187 women after the application of criteria (i) to (iii); and (vi) data collected after April 2017, because the reference materials for glucose measurement had been replaced.

**Fig 2:**
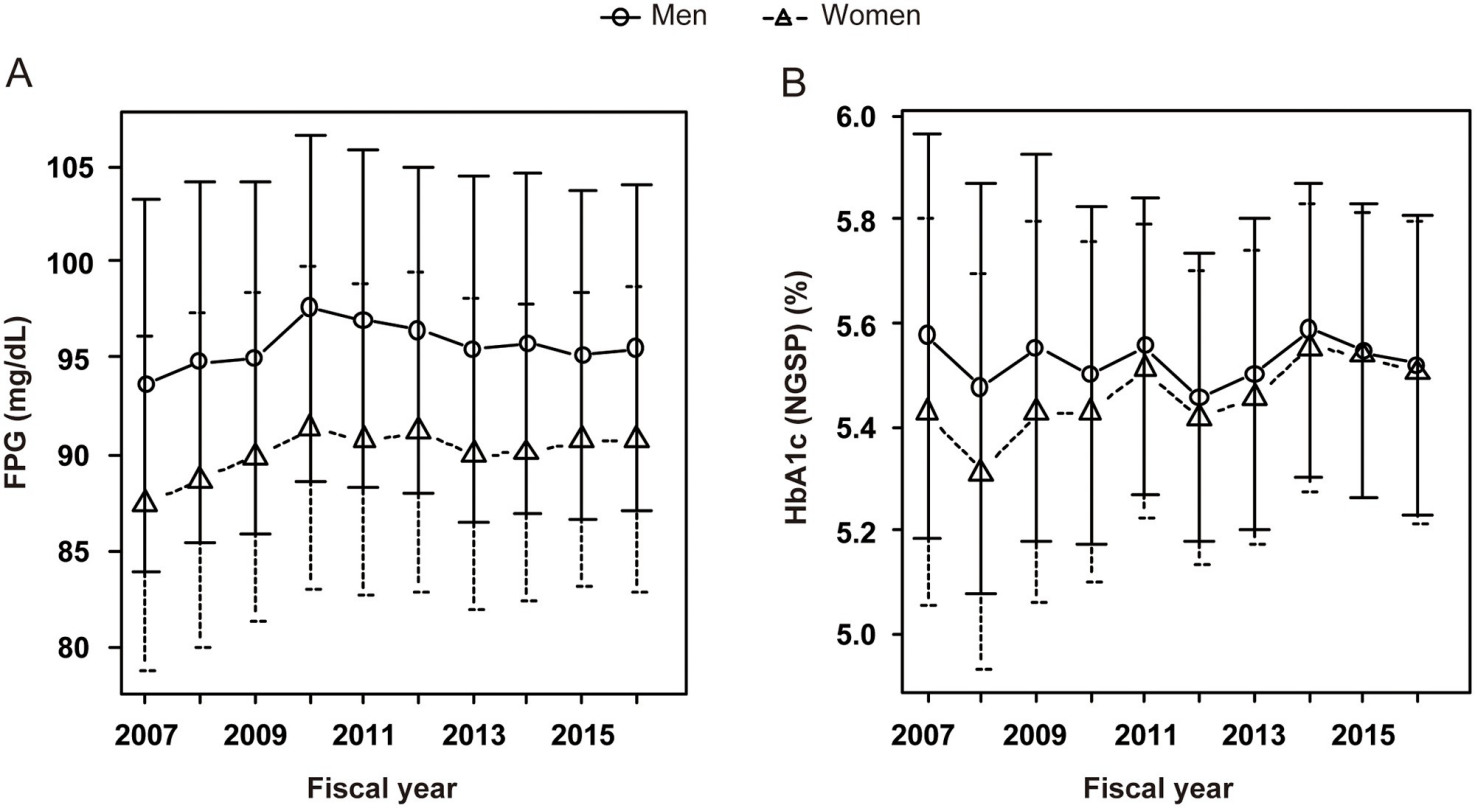
Results of the exploratory analysis. The annual trends in fasting plasma glucose (FPG) concentration (panel A) and glycated hemoglobin (HbA1c) level (panel B) before adjustment for background variables. Circles and solid lines represent the mean values in men and triangles and dashed lines represent the mean values in women. Error bars represent the standard deviation. See Materials and Methods for details.

We evaluated the remaining 16,370 men and 11,695 women during the initial exploratory analysis (Fig 2).

### Clinical and laboratory assessment

Clinical and laboratory parameters were assessed as described in detail previously [4]. Briefly, a physical examination was conducted to determine the body height (BH), body weight (BW), waist circumference (WC), blood pressure (BP), and heart rate (HR) of the participants after an overnight fast. Then, their body mass index (BMI) was calculated. WC was measured at the level of the umbilicus, according to the Japan Society for the Study of Obesity recommendations [5]. Information regarding the use of medication and visits to the hospital or family physician was obtained using questionnaires. Blood samples were obtained to measure FPG, fasting serum immunoreactive insulin (F-IRI), HbA_1c_, serum lipid concentrations, hemoglobin (Hb) level, and red blood cell (RBC) count. FPG was measured using auto-analyzers equipped with a glucose oxidase-immobilized membrane and a hydrogen peroxide-sensing electrode (GA-1160 and GA-1171; Arkray, Kyoto, Japan). F-IRI was measured with auto-analyzers using a chemiluminescent enzyme immunoassay (Lumipulse and Lumipulse Presto; Fujirebio, Tokyo, Japan). HbA_1c_ was measured using high-performance liquid chromatography (ADAMS; Arkray). The HbA_1c_ (%) values are the National Glycohemoglobin Standardization Program (NGSP) values. The formula used for the conversion of HbA_1c_ (Japan Diabetes Society) to HbA_1c_ (NGSP) [6, 7] was:

$${\text{HbA}}_{1\text{c}}\left(\text{NGSP}\right)\;\left(\%\right)=1.02\;\times {\;\text{HbA}}_{1\text{c}}\left(\text{Japan}\;\text{Diabetes}\;\text{Society}\right)\;\left(\%\right)+0.25\%$$

The homeostasis model assessment of insulin resistance (HOMA-IR) and insulin secretion (HOMA-β) were calculated using the following formulae [8]:

$$\text{HOMA-IR}=\text{F-IRI}\;\left(\mu \text{IU}/\text{mL}\right)\;\times \;\text{FPG}\;\left(\text{mg}/\text{dL}\right)/405,$$

and

$$\text{HOMA-β}=360\;\times \;\text{F-IRI}\;\left(\mu \text{IU}/\text{mL}\right)/\left(\text{FPG}-63\right)\;\left(\text{mg}/\text{dL}\right)$$

The serum total adiponectin concentration was measured using a latex particle-enhanced turbidimetric immunoassay (Human Adiponectin Latex Kit; Otsuka Pharmaceuticals, Tokyo, Japan) [9]. The serum concentrations of total cholesterol and triglyceride were determined using enzymatic methods (Determiner-L; Kyowa Medex, Tokyo, Japan). The assay systems for the serum concentrations of low-density lipoprotein-cholesterol (LDL-C) and high-density lipoprotein-cholesterol (HDL-C) were replaced during the study period: LDL-EX and HDL-EX (Denka, Tokyo, Japan) were used before June 2011 and Determiner and Metabolead (Kyowa Medex) were used after July 2011. The correlation between the measurements using the old and new methods was not close; therefore, the data are presented in the tables for information only, and they are not discussed further. The serum activities of aspartate aminotransferase (AST) and alanine aminotransferase (ALT) were measured with an Iatro LQ auto-analyzer (LSI Medicine Corporation, Tokyo, Japan) using a kinetic method. The serum activity of γ-glutamyltranspeptidase (γ-GTP) was measured with Iatro LQ (LSI Medience Corporation), Cicaliquid (Kanto Chemical, Tokyo, Japan), and Labofit (Kanto Chemical) auto-analyzers using a colorimetric method. Hemoglobin (Hb) concentration was measured using the cyanmethemoglobin method (LH780; Beckman Coulter, Brea, CA, USA). Throughout the study period, the biochemical analyzers were rigorously calibrated using reference materials that were certified by a national agency to be traceable to international primary reference materials. The same batches of calibrators for plasma glucose and IRI were used throughout the study period, and batch-to-batch consistency of the HbA1c calibrator was confirmed by a national agency.

### Statistical analyses

For testing of the statistical hypothesis, data for non-diabetic individuals collected during the 2007–2008 fiscal year were pair-matched with those collected during the 2015–2016 fiscal year with respect to sex, age, smoking status, Hb, and RBC count (1:1 nearest-neighbor matching using a caliper of 0.2) (Fig 1). For each variable, a standard mean difference (SMD) of < 0.1 was confirmed (Tables 1 and 2). Of the 8,417 men and 5,839 women, 3,140 men and 2,048 women were pair-matched and their data were further evaluated. Data are presented as mean (standard deviation (SD)). The differences in the mean values were evaluated using Welch’s *t*-test, and the chi-square test of independence was used to evaluate smoking status. The power of the study was set at 80%, with a significance level of 0.05. Statistical analyses were undertaken using R (versions 3.5.2 and 4.0.2) and R commander GUI (versions 2.5–1 and 2.7–0) with the EZR plugin (version 1.40 and 1.53) [10].

**Table 1:** Clinical characteristics and biochemical parameters of men without diabetes mellitus before and after pair-matching.

	Before matching				After matching (caliper = 0.2)			
	2007~2008	2015~2016	*P* value	*SMD	2007~2008	2015~2016	*P* value	*SMD
Men								
*N*	6321	2096			1570	1570		
Age	51.95 (11.15)	47.72 (10.94)	<0.001	0.383	47.96 (9.99)	47.94 (9.99)	0.94	0.003
Hb (g/dL)	15.11 (1.03)	15.14 (1.04)	0.304	0.026	15.16 (0.83)	15.14 (0.85)	0.479	0.025
RBC (10^6^/μL)	4.72 (0.40)	4.97 (0.41)	<0.001	0.635	4.90 (0.34)	4.91 (0.34)	0.548	0.021
Never smoker (%)	1957 (31.0)	755 (36.0)			548 (34.9)	548 (34.9)		
Ex-smoker (%)	2427 (38.4)	786 (37.5)	NA	0.118	600 (38.2)	600 (38.2)	1	<0.001
Current smoker (%)	1937 (30.6)	555 (26.5)			422 (26.9)	422 (26.9)		
BH (cm)	170.12 (6.03)	171.85 (6.00)	<0.001	0.288	170.95 (6.17)	172.01 (5.95)	<0.001	0.174
BW (kg)	68.26 (9.61)	69.94 (10.65)	<0.001	0.165	69.72 (10.07)	69.72 (10.21)	0.985	0.001
BMI	23.56 (2.85)	23.65 (3.13)	0.22	0.03	23.83 (2.96)	23.54 (3.03)	**(0.007)	0.096
WC (cm)	85.59 (7.83)	84.58 (8.60)	<0.001	0.123	85.83 (7.94)	84.30 (8.35)	<0.001	0.187
SBP (mmHg)	126.41 (14.94)	120.53 (14.45)	<0.001	0.4	126.30 (14.86)	120.25 (14.15)	<0.001	0.417
DBP (mmHg)	79.42 (10.02)	75.20 (10.95)	<0.001	0.402	79.41 (10.05)	75.34 (10.69)	<0.001	0.392
HR (bpm)	73.02 (10.70)	63.61 (9.63)	<0.001	0.925	73.87 (11.13)	63.42 (9.16)	<0.001	1.025
AST (U/L)	23.90 (10.54)	23.08 (8.57)	0.001	0.085	23.32 (10.38)	23.17 (8.58)	0.653	0.016
ALT (U/L)	26.00 (18.44)	25.34 (16.54)	0.142	0.038	27.47 (19.99)	25.06 (16.58)	<0.001	0.131
γ-GTP (U/L)	53.76 (65.37)	48.11 (55.82)	<0.001	0.093	50.14 (50.28)	48.58 (54.04)	0.401	0.03
Tcho (mg/dL)	211.27 (32.23)	204.65 (33.92)	<0.001	0.2	212.29 (34.03)	204.86 (32.77)	<0.001	0.222
LDL-C (mg/dL)	127.80 (30.79)	126.81 (30.80)	0.203	0.032	130.56 (31.56)	126.69 (29.82)	<0.001	0.126
HDL-C (mg/dL)	58.51 (13.73)	59.69 (14.88)	0.001	0.082	56.29 (12.45)	60.40 (14.90)	<0.001	0.3
log TG (log mg/dL)	4.76 (0.53)	4.67 (0.57)	<0.001	0.175	4.78 (0.55)	4.66 (0.57)	<0.001	0.214
FPG (mg/dL)	94.01 (9.59)	95.34 (8.51)	<0.001	0.146	93.71 (9.59)	95.31 (8.60)	<0.001	0.175
HbA1c NGSP (%)	5.54 (0.40)	5.53 (0.29)	0.228	0.033	5.52 (0.39)	5.52 (0.28)	0.59	0.019
log F-IRI (log μIU/mL)	1.67 (0.53)	1.72 (0.55)	<0.001	0.091	1.72 (0.52)	1.70 (0.54)	0.131	0.054
log HOMA-β	4.18 (0.54)	4.17 (0.51)	0.45	0.019	4.24 (0.51)	4.14 (0.50)	<0.001	0.19
log HOMA-IR	0.21 (0.57)	0.27 (0.58)	<0.001	0.111	0.25 (0.56)	0.24 (0.58)	0.61	0.018
log adiponectin (log μg/mL)	1.97 (0.46)	2.00 (0.43)	0.001	0.082	1.93 (0.45)	2.00 (0.42)	<0.001	0.182

**Table 2:** Clinical characteristics and biochemical parameters of women without diabetes mellitus before and after pair-matching.

	Before matching				After matching (caliper = 0.2)			
	2007~2008	2015~2016	*P* value	SMD	2007~2008	2015~2016	*P* value	*SMD
Women								
*N*	4282	1557			1024	1024		
Age	49.74 (11.68)	48.18 (11.79)	<0.001	0.133	48.37 (10.74)	48.34 (10.80)	0.946	0.003
Hb (g/dL)	13.18 (1.16)	13.07 (1.15)	0.001	0.099	13.13 (0.98)	13.10 (0.97)	0.632	0.021
RBC (10^6^/μL)	4.25 (0.33)	4.44 (0.34)	<0.001	0.571	4.37 (0.28)	4.37 (0.29)	0.672	0.019
Never smoker (%)	3618 (84.5)	1212 (77.8)			900 (87.9)	900 (87.9)		
Ex-smoker (%)	327 (7.6)	223 (14.3)	<0.001	0.216	87 (8.5)	87 (8.5)	1	<0.001
Current smoker (%)	337 (7.9)	122 (7.8)			37 (3.6)	37 (3.6)		
BH (cm)	157.75 (5.55)	158.43 (5.46)	<0.001	0.124	158.12 (5.38)	158.56 (5.35)	0.065	0.082
BW (kg)	52.48 (7.72)	53.05 (8.38)	0.014	0.071	53.27 (8.00)	52.76 (8.07)	0.151	0.063
BMI	21.09 (2.99)	21.13 (3.16)	0.663	0.013	21.32 (3.14)	20.98 (3.03)	**(0.014)	0.108
WC (cm)	77.32 (8.88)	77.21 (8.94)	0.677	0.012	77.59 (8.86)	76.66 (8.78)	0.018	0.105
SBP (mmHg)	119.52 (16.57)	114.52 (15.61)	<0.001	0.311	119.00 (16.03)	113.81 (15.32)	<0.001	0.331
DBP (mmHg)	73.87 (10.55)	70.18 (10.60)	<0.001	0.35	73.58 (10.38)	69.91 (10.29)	<0.001	0.355
HR (bpm)	76.11 (11.45)	64.23 (9.79)	<0.001	1.115	77.20 (11.32)	63.83 (9.65)	<0.001	1.272
AST (U/L)	20.53 (8.84)	19.60 (6.47)	<0.001	0.121	19.94 (6.99)	19.83 (6.81)	0.715	0.016
ALT (U/L)	16.98 (11.35)	15.73 (9.71)	<0.001	0.119	16.76 (11.06)	15.87 (10.17)	0.057	0.084
γ-GTP (U/L)	23.92 (26.81)	22.35 (20.99)	0.037	0.065	22.79 (23.00)	22.14 (19.57)	0.486	0.031
Tcho (mg/dL)	215.30 (35.99)	209.43 (37.22)	<0.001	0.161	214.19 (36.09)	209.64 (36.92)	0.005	0.125
LDL-C (mg/dL)	119.90 (32.02)	122.91 (33.15)	0.002	0.093	120.23 (32.23)	122.95 (32.65)	0.058	0.084
HDL-C (mg/dL)	72.17 (16.40)	75.33 (16.58)	<0.001	0.192	71.47 (16.45)	76.27 (16.27)	<0.001	0.294
log TG (log mg/dL)	4.35 (0.47)	4.28 (0.47)	<0.001	0.162	4.36 (0.46)	4.24 (0.45)	<0.001	0.248
FPG (mg/dL)	87.87 (8.77)	90.73 (7.80)	<0.001	0.345	87.58 (8.72)	90.59 (7.94)	<0.001	0.36
HbA1c NGSP (%)	5.38 (0.38)	5.52 (0.29)	<0.001	0.403	5.41 (0.36)	5.51 (0.29)	<0.001	0.312
log F-IRI (log μIU/mL)	1.51 (0.48)	1.58 (0.50)	<0.001	0.142	1.55 (0.46)	1.54 (0.48)	0.871	0.007
log HOMA-β	4.25 (0.49)	4.18 (0.46)	<0.001	0.138	4.30 (0.48)	4.15 (0.45)	<0.001	0.313
log HOMA-IR	-0.02 (0.53)	0.08 (0.54)	<0.001	0.192	0.01 (0.51)	0.04 (0.53)	0.168	0.061
log adiponectin (log μg/mL)	2.47 (0.46)	2.46 (0.45)	0.596	0.016	2.45 (0.46)	2.49 (0.44)	**(0.015)	0.108

### Ethics approval

The study was approved by the Sumitomo Hospital ethics committee (number 30–28) and conducted in accordance with the ethics standards of the responsible committee on human experimentation (institutional and national) and with the Helsinki Declaration of 1964 and its later amendments. Written informed consent was obtained from the participants before their check-up. Individuals were informed that their participation was voluntary, and that they could withdraw consent to participate at any time.

## Results

We first performed an exploratory analysis to better understand the basic characteristics of the non-diabetic participants who visited our center for the first time (Fig 1). Although their background characteristics, such as age, smoking status, and the presence or absence of anemia, which could affect HbA_1c_ level, were not matched between fiscal years, the mean FPG and HbA_1c_ of the women seemed to have increased during this period, as suggested by Lilja *et al*. (an increase in FPG) [11], Magliano *et al*. (an increase in FPG) [12], and Cheng *et al*. (an increase in HbA_1c_) [13] (Fig 2).

Next, we undertook a matched-pair analysis of the data collected during the 2007–2008 fiscal year and the 2015–2016 fiscal year with respect to sex, age, smoking status, Hb level, and RBC count for statistical hypothesis testing (Fig 1; see Materials and Methods). Matching for Hb level and RBC count was performed to limit the confounding effect of erythrocyte kinetics on HbA_1c_. For each variable, a SMD < 0.1 was confirmed. Totals of 3,140 men and 2,048 women were selected using the matched-pair analysis. The men had a mean age of 48.0 years, 34.9% had never smoked, 38.2% were ex-smokers, and 26.9% were current smokers (Table 1). The women had a mean age of 48.4 years, 87.9% had never smoked, 8.5% were ex-smokers, and 3.6% were current smokers (Table 2).

Non-diabetic men who underwent medical check-ups for the first time during 2007–2008 were pair-matched to those who underwent check-ups during 2015–2016 with respect to sex, age, smoking status, hemoglobin (Hb) level, and red blood cell (RBC) count. Data are presented as mean (SD). The differences in the mean values were evaluated using Welch’s *t*-test, and the chi-square test of independence was used to evaluate smoking status. The power of the study was set at 80%, with a significance level of 0.05. *SMD: standard mean difference. **Did not achieve the sample size yielded by the power analysis. The assay systems for serum LDL-C and HDL-C were replaced during the study period and the correlation between the measurements made using the old and new methods was not close. Therefore, we present the data for information and do not discuss it in the text (see Materials and Methods). Abbreviations: BH, body height; BW, body weight; BMI, body mass index; WC, waist circumference; SBP, systolic blood pressure; DBP, diastolic blood pressure; HR, heart rate; AST, aspartate aminotransferase; ALT, alanine aminotransferase; γ-GTP, γ-glutamyl transpeptidase; Tcho, total cholesterol; LDL-C, low-density lipoprotein cholesterol; HDL-C, high-density lipoprotein cholesterol; TG, triglycerides; FPG, fasting plasma glucose; HbA1c, glycated hemoglobin; F-IRI, fasting immunoreactive insulin; HOMA-β, homeostasis model assessment of insulin secretion; and HOMA-IR, homeostasis model assessment of insulin resistance.

Non-diabetic women who underwent medical check-ups for the first time during 2007–2008 were pair-matched to those who underwent check-ups during 2015–2016 with respect to sex, age, smoking status, Hb level, and RBC count. Data are presented as mean (SD). The differences in the mean values were evaluated using Welch’s *t*-test, and the chi-square test of independence was used to evaluate smoking status. The power of the study was set at 80%, with a significance level of 0.05. *SMD: standard mean difference. **Did not achieve the sample size yielded by the power analysis. The assay systems for serum LDL-C and HDL-C were replaced during the study period and the correlation between the measurements made using the old and new methods was not close. Therefore, we present the data for information and do not discuss it in the text (see Materials and Methods). Abbreviations: BH, body height; BW, body weight; BMI, body mass index; WC, waist circumference; SBP, systolic blood pressure; DBP, diastolic blood pressure; HR, heart rate; AST, aspartate aminotransferase; ALT, alanine aminotransferase; γ-GTP, γ-glutamyl transpeptidase; Tcho, total cholesterol; LDL-C, low-density lipoprotein cholesterol; HDL-C, high-density lipoprotein cholesterol; TG, triglycerides; FPG, fasting plasma glucose; HbA1c, glycated hemoglobin; F-IRI, fasting immunoreactive insulin; HOMA-β, homeostasis model assessment of insulin secretion; and HOMA-IR, homeostasis model assessment of insulin resistance.

WC significantly decreased from 2007–2008 to 2015–2016 in both sexes: the difference in the mean value (Δ) was −1.5 cm (*P* < 0.001) in men and −0.9 cm (*P* = 0.02) in women (Tables 1 and 2). The serum concentration of total adiponectin increased in men. The adiponectin concentration in women and the BMI of both sexes did not achieve the calculated minimum sample size.

The BP, HR, serum total cholesterol (Tcho), and serum triglyceride (TG) significantly decreased in both sexes (Tables 1 and 2). Specifically, in men, Δ systolic BP (SBP) was −6 mmHg (*P* < 0.001), Δ diastolic BP (DBP) was −4 mmHg (*P* < 0.001), Δ HR was −10.5 bpm (*P* < 0.001), Δ Tcho was −7.4 mg/dL (*P* < 0.001), and Δ TG was −13.5 mg/dL (*P* < 0.001). In women, Δ SBP was −5 mmHg (*P* < 0.001), Δ DBP was −4 mmHg (*P* < 0.001), Δ HR was −13 bpm (*P* < 0.001), Δ Tcho was −4.6 mg/dL (*P* = 0.005), and Δ TG was −8.8 mg/dL (*P* < 0.001).

**Fig 3:**
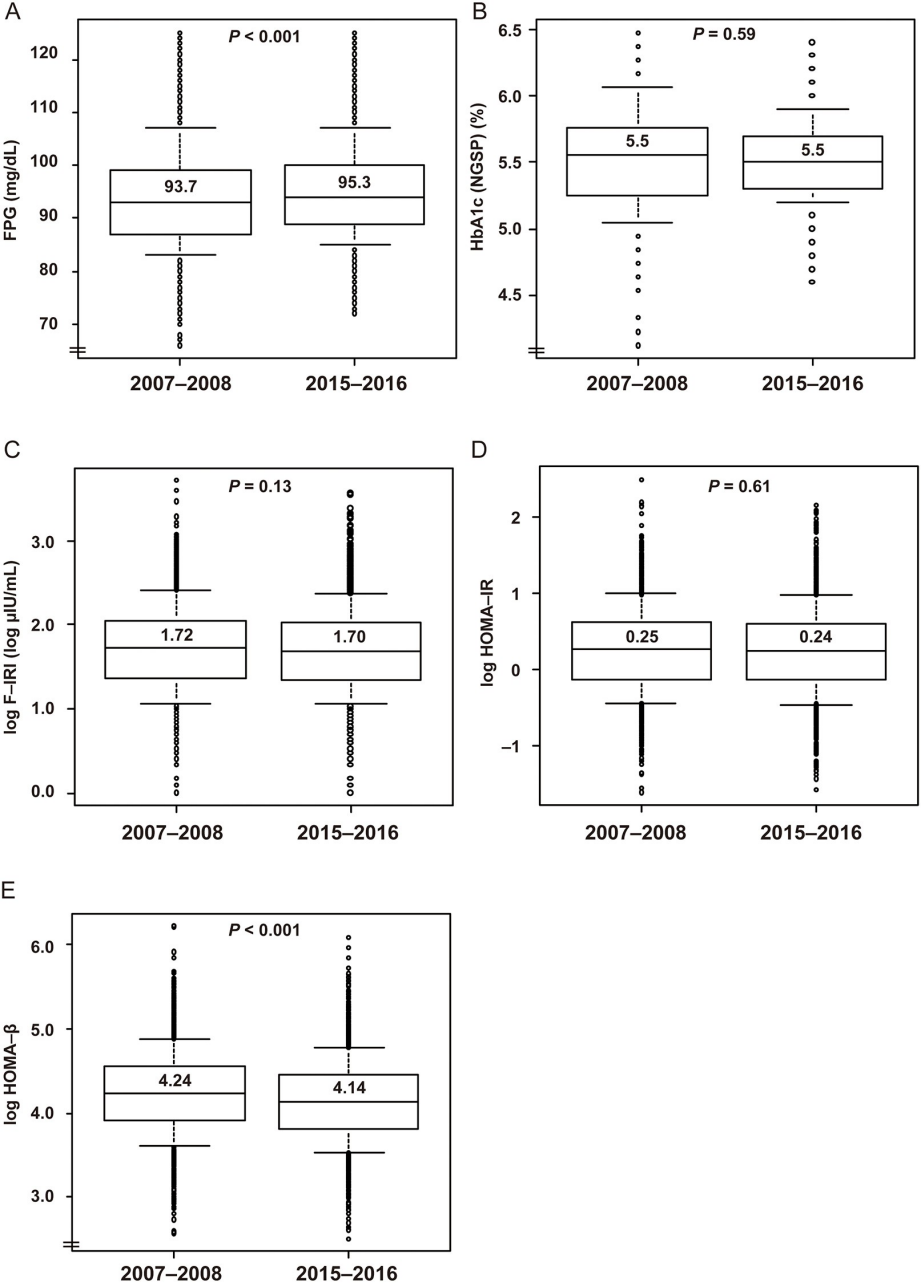
Box-and-whisker plots for FPG, HbA1c, F-IRI, HOMA-IR, and HOMA-β in men. Whiskers indicate the 10th and 90th percentiles. The Y axes do not start at zero in panels A, B, D, or E. F-IRI, HOMA-IR, and HOMA-β (Y-axes) are plotted on a natural logarithmic scale. Abbreviations: FPG, fasting plasma glucose; HbA1c, glycated hemoglobin; F-IRI, fasting immunoreactive insulin; HOMA-β, homeostasis model assessment of insulin secretion; and HOMA-IR, homeostasis model assessment of insulin resistance. *P*-values were determined using Welch’s *t*-test. *N* = 1,570 for each group.

**Fig 4:**
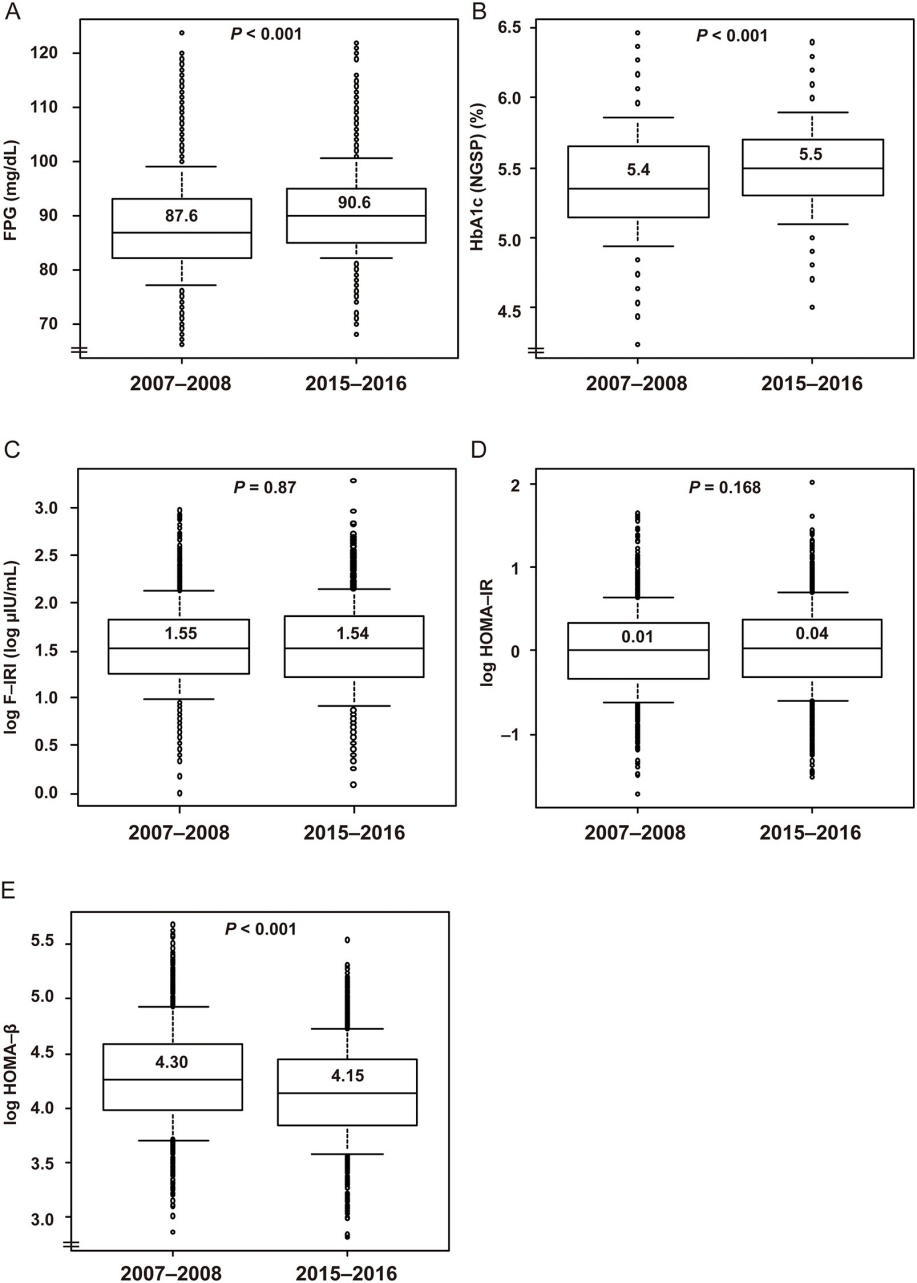
Box-and-whisker plots for FPG, HbA1c, F-IRI, HOMA-IR, and HOMA-β in women. Whiskers indicate the 10th and 90th percentiles. The Y axes do not start at zero in panels A, B, D, or E. F-IRI, HOMA-IR, and HOMA-β (Y-axes) are plotted on a natural logarithmic scale. Abbreviations: FPG, fasting plasma glucose; HbA1c, glycated hemoglobin; F-IRI, fasting immunoreactive insulin; HOMA-β, homeostasis model assessment of insulin secretion; and HOMA-IR, homeostasis model assessment of insulin resistance. *P*-values were determined using Welch’s *t*-test. *N* = 1,024 for each group.

In sharp contrast to WC, BP, HR, and lipid concentrations, FPG concentration increased in both sexes and HbA_1c_ increased in women. Specifically, in men, Δ FPG was +1.6 mg/dL (*P* < 0.001) and Δ HbA1c was ±0% (*P* = 0.6) (Table 1, Fig 3). In women, Δ FPG was +3.0 mg/dL (*P* <0.001) and Δ HbA1c was +0.1% (*P* <0.001) (Table 2, Fig 4). Thus, there were larger changes in these parameters in women than in men. F-IRI and the derived Δ HOMA-IR did not significantly change in either sex. In contrast, Δ HOMA-β significantly decreased (−6.6 in men (*P* < 0.001) and −10.3 in women (*P* <0.001)), which suggested that blunted secretion of insulin may have had a causal role in the increase in FPG concentration.

## Discussion

In this large-scale, retrospective analysis, we have shown an increase in FPG concentration in non-diabetic individuals that coincided with the implementation of the Japanese National Intervention Program for metabolic syndrome. Studies conducted in women living in northern Sweden between 1990 and 2009 and in Mauritius between 1987 and 2009 also showed increases in the FPG concentrations of non-diabetic populations [11, 12]. In all three studies, the entire distribution of FPG was shifted upwards. Thus, the increase in the FPG of non-diabetic individuals may represent a global trend and suggests a widespread impact of environmental changes. Interestingly, in the present study, the HbA_1c_ level of women showed a more pronounced upward shift at the lower end of its distribution than at the upper end. A study conducted between 1988 and 2006 in the USA showed a similar trend [13].

Surprisingly, the increase in FPG concentration was not associated with an increase in obesity. Therefore, lifestyle or environmental factors could have influenced glucose metabolism through obesity-independent mechanisms. Notably, HOMA-β decreased during the study period, suggesting that the obesity-independent changes in glucose regulation involve alterations in pancreatic β-cell function. The importance of pancreatic β-cell function for FPG has been previously established in other systems [14–16]. A recent study that used a pancreatic islet xenotransplantation system showed that pancreatic islets are the *bona fide* “glucostat” that determine the “glycemic set point” for the whole body [17]. In 2006, a hypothesis was proposed that insulin secretion is affected by the social environment, but the data generated were severely confounded by increases in obesity and insulin resistance [18]. Thus, the current study is the first to provide objective data that are consistent with this hypothesis.

The regulation of the glycemic set point is an emerging field of research [17, 19, 20]. The participants in the present study did not have diabetes mellitus or high serum lipid concentrations. Thus, it is unlikely that glucotoxicity or lipotoxicity played a part in the modification of pancreatic β-cell function, although we cannot rule out the contribution of specific lipid molecules [21, 22].

HOMA-IR is computed by multiplying FPG by F-IRI. Hence, HOMA-IR might have increased, but did not. The assessment of insulin resistance can be confounded by mild polycythemia; therefore, the pair-matching procedure for Hb level and RBC count might have led to an underestimation of the change in insulin resistance. The increase in the log HOMA-IR value became significant when pair-matching for Hb level and RBC count was not performed (1.24 (SD 1.75) during 2007–2008 *vs*. 1.31 (SD 1.79) during 2015–2016 for men (*P* = 0.001); 0.98 (SD 1.68) during 2007–2008 *vs*. 1.08 (SD 1.72) during 2015–2016 for women (*P* < 0.001)). In addition, HOMA-IR is known to underestimate the level of insulin resistance [29]. If the insulin resistance of the participants was in fact increased, this might have exacerbated the decrease in insulin secretion and the increase in FPG.

It is possible that the relatively small variations in FPG and HbA_1c_ may have been the result of measurement bias. However, the following observations make this less likely: (i) sex differences; (ii) parallelism between FPG and HbA_1c_; (iii) individuals at the lower ends of the distributions of FPG (in both sexes) and HbA_1c_ (in women) showed larger increases than individuals at the upper ends, as shown by Cheng and colleagues [13]. Our single-center analysis was robust, because we measured the parameters in all the samples at a single laboratory, which minimizes measurement bias during the identification of small, but significant changes, especially when the standardization and harmonization of the insulin immunoassays are still in progress [23]. According to a study by Ito of 15,191 Japanese individuals, HbA_1c_ (%) was equal to 0.0334 × FPG (mg/dL) + 2.26 (*R*^*2*^ = 0.7321, *P* < 0.0001) [24]. Therefore, the Δ FPG of 3 mg/dL identified in the present study is consistent with the Δ HbA_1c_ of 0.1%.

An increase of 0.1% in HbA_1c_ may not have a significant impact on individual health. However, the same Δ HbA_1c_ of +0.1% has been shown to have significant effects on public health: it increases the risk of ventricular fibrillation in non-diabetic individuals by 1.4-fold, independent of concomitant cardiovascular risk factors [25].

In recent years, male sex has come to be regarded as a risk factor for the development of T2D [26]. Therefore, it may seem surprising that women showed larger changes than men in the present study. However, Gale and Gillespie reported that many more women than men had T2D in the first half of the twentieth century [27]. Thus, it seems that the prevailing socio-environmental conditions may dictate which sex is more susceptible to metabolic derangements over time.

The National Health and Nutrition Survey of Japan does not involve measurements of either FPG or F-IRI [28]. Furthermore, the National Health and Nutrition Survey combines data from 47 prefectures, each of which has a unique social environment. The maximum difference in the mean BMI among the prefectures was 2.1 kg/m^2^ in 2016 [28], which could be large enough to obscure moderate changes in metabolic parameters. Thus, our rigorous approach permitted the identification of a previously unknown impact of lifestyle or environmental factors on glucose metabolism.

The present retrospective observational single-center study had the following inherent limitations: (i) selection bias, owing to recruitment at a single center; (ii) selection bias, owing to a “healthy adherer” effect; (iii) the possibility of measurement bias; (iv) the possibility of there being unknown confounding factors; (v) small, although significant, differences, owing to the large sample size. In addition, HOMA indices are surrogate measures [8, 29]. More elaborate assays have been developed for the assessment of insulin secretion and sensitivity. In particular, measurement of the serum concentration of C-peptide would have helped us to estimate the contribution of hepatic insulin extraction to the serum insulin concentration [30]. Matthews and the other scholars who developed the HOMA indices have suggested that they are appropriate for use in large epidemiological studies, including in healthy populations, as in the present study [29]. It is also important to note that the statistical matching procedure has its own limitations, and the sample selected by matching may not be fully representative of the pre-matching sample. Furthermore, the existence of the National Intervention Program might have affected the likelihood of each member of the general population attending a hospital-based medical check-up, which might have generated additional biases.

We suspect that were it not for the National Intervention Program, WC might have increased during the study period, and we might have attributed the deterioration in glucose status to the worsening of visceral obesity. Therefore, we believe that the existence of the program has highlighted the significance of both visceral obesity-dependent and independent pathways for glucose metabolism. Considering that an increase in FPG in non-diabetic individuals has also been shown over time in other countries [11, 12], globally relevant environmental factors, such as exposure to endocrine disruptors, may be mediators of the latter pathway. Future studies should aim to elucidate the underlying mechanism.

It is challenging to provide a mechanistic explanation of the effects of environmental factors on T2D, the progression from normal glucose tolerance to T2D is insidious, and multiple pathogenic factors can have secondary effects. Therefore, research in non-diabetic populations, as in the present study, may advance understanding of the etiology of T2D.

## Conclusions

Non-diabetic people have shown an unexpected increase in their glycemic set points during recent years. Our unique approach has established a framework for future epidemiological and basic studies regarding obesity-independent environmental effects on glucose metabolism and the mechanisms involved.

## Supporting information

S1 Table Clinical characteristics and biochemical parameters of men without diabetes mellitus after pair-matching. The results of 1:1 nearest-neighbor matching performed using a caliper of 0.25 were compared to those using a caliper of 0.2. Similar results were obtained. ** Did not achieve the sample size yielded by the power analysis.
(PDF)

S2 Table Clinical characteristics and biochemical parameters of women without diabetes mellitus after pair-matching. The results of 1:1 nearest-neighbor matching performed using a caliper of 0.25 were compared to those using a caliper of 0.2. Essentially similar results were obtained. ** Did not achieve the sample size yielded by the power analysis.
(PDF)

S3 Table Clinical characteristics and biochemical parameters of men without diabetes mellitus before and after pair-matching. Unlike in Table 1, the data collected at the second and later visits by the same individuals were NOT omitted. This amendment caused an increase in the mean age of the participants from 2007–2008 to 2015–2016 before matching. After matching with respect to age, in addition to smoking status, Hb, and RBC, the risk of comparing the same individuals at different ages was abolished. Similar results to those shown in Table 1 were obtained, except that log F-IRI and log HOMA-IR significantly decreased from 2007–2008 to 2015–2016. The relatively large sample size allowed the BMI data to achieve the sample size yielded by the power analysis.
(PDF)

S4 Table Clinical characteristics and biochemical parameters of women without diabetes mellitus before and after pair-matching. Unlike in Table 2, the data collected at the second and later visits by the same individuals were NOT omitted. This amendment caused an increase in the mean age from 2007–2008 to 2015–2016 before matching. After matching with respect to age, in addition to smoking status, Hb, and RBC, the risk of comparing the same individuals at different ages was abolished. Similar results to those shown in Table 2 were obtained, except that BH significantly increased and γ-GTP significantly decreased from 2007–2008 to 2015–2016. The relatively large sample size permitted BW, BMI, ALT, and log adiponectin to achieve the sample size yielded by the power analysis. ** Did not achieve the sample size yielded by the power analysis.
(PDF)
