# Supplementary material for: Increase in glycemic set point, alongside a decrease in waist circumference, in the non-diabetic population during the Japanese National Intervention Program for metabolic syndrome: A single-center, large-scale, matched-pair analysis
Source: PLoS One. 2022 Aug 10;17(8):e0268450. doi: 10.1371/journal.pone.0268450 (PMC9365144; doi:10.1371/journal.pone.0268450)
Supplement: S1 Table — The results of 1:1 nearest-neighbor matching performed using a caliper of 0.25 were compared to those using a caliper of 0.2. Similar results were obtained. ** Did not achieve the sample size yielded by the power analysis. (PDF) [file pone.0268450.s001.pdf]

S1 Table

	After matching (caliper = 0.2)				After matching (caliper = 0.25)			
	2007～2008	2015～2016	*P* value	SMD	2007～2008	2015～2016	*P* value	SMD
<u>Men</u>								
N	1570	1570			1620	1620		
Age	47.96 (9.99)	47.94 (9.99)	0.94	0.003	47.92 (10.13)	47.89 (10.14)	0.913	0.004
Hb (g/dL)	15.16 (0.83)	15.14 (0.85)	0.479	0.025	15.16 (0.85)	15.14 (0.86)	0.476	0.025
RBC (10^6/μL)	4.90 (0.34)	4.91 (0.34)	0.548	0.021	4.90 (0.34)	4.91 (0.34)	0.427	0.028
Never smoker (%)	548 (34.9)	548 (34.9)			571 (35.2)	571 (35.2)		
Ex-smoker (%)	600 (38.2)	600 (38.2)	1	<0.001	617 (38.1)	617 (38.1)	1	<0.001
Current smoker (%)	422 (26.9)	422 (26.9)			432 (26.7)	432 (26.7)		
BH (cm)	170.95 (6.17)	172.01 (5.95)	<0.001	0.174	170.94 (6.13)	172.00 (5.92)	<0.001	0.176
BW (kg)	69.72 (10.07)	69.72 (10.21)	0.985	0.001	69.70 (10.00)	69.73 (10.23)	0.933	0.003
BMI	23.83 (2.96)	23.54 (3.03)	**(0.007)	0.096	23.82 (2.94)	23.54 (3.01)	**(0.007)	0.095
WC (cm)	85.83 (7.94)	84.30 (8.35)	<0.001	0.187	85.82 (7.90)	84.31 (8.35)	<0.001	0.186
SBP (mmHg)	126.30 (14.86)	120.25 (14.15)	<0.001	0.417	126.32 (14.96)	120.22 (14.20)	<0.001	0.418
DBP (mmHg)	79.41 (10.05)	75.34 (10.69)	<0.001	0.392	79.39 (10.09)	75.27 (10.71)	<0.001	0.396
HR (bpm)	73.87 (11.13)	63.42 (9.16)	<0.001	1.025	73.79 (11.05)	63.33 (9.15)	<0.001	1.03
AST (U/L)	23.32 (10.38)	23.17 (8.58)	0.653	0.016	23.26 (10.31)	23.16 (8.66)	0.765	0.011
ALT (U/L)	27.47 (19.99)	25.06 (16.58)	<0.001	0.131	27.33 (19.77)	25.05 (16.73)	<0.001	0.125
γ-GTP (U/L)	50.14 (50.28)	48.58 (54.04)	0.401	0.03	50.01 (50.40)	48.54 (54.35)	0.425	0.028
Tcho (mg/dL)	212.29 (34.03)	204.86 (32.77)	<0.001	0.222	212.57 (33.87)	205.05 (33.72)	<0.001	0.222
LDL-C (mg/dL)	130.56 (31.56)	126.69 (29.82)	<0.001	0.126	130.81 (31.53)	126.96 (30.81)	<0.001	0.124
HDL-C (mg/dL)	56.29 (12.45)	60.40 (14.90)	<0.001	0.3	56.27 (12.48)	60.44 (14.88)	<0.001	0.304
log TG (log mg/dL)	4.78 (0.55)	4.66 (0.57)	<0.001	0.214	4.78 (0.55)	4.66 (0.57)	<0.001	0.226
FPG (mg/dL)	93.71 (9.59)	95.31 (8.60)	<0.001	0.175	93.70 (9.50)	95.28 (8.58)	<0.001	0.174
HbA1c NGSP (%)	5.52 (0.39)	5.52 (0.28)	0.59	0.019	5.52 (0.39)	5.52 (0.28)	0.536	0.022
log F-IRI (log μIU/mL)	1.72 (0.52)	1.70 (0.54)	0.131	0.054	1.73 (0.51)	1.69 (0.54)	0.071	0.063
log HOMA-β	4.24 (0.51)	4.14 (0.50)	<0.001	0.19	4.24 (0.51)	4.14 (0.51)	<0.001	0.196
log HOMA-IR	0.25 (0.56)	0.24 (0.58)	0.61	0.018	0.26 (0.56)	0.24 (0.58)	0.434	0.027
log adiponectin (log μg/mL)	1.93 (0.45)	2.00 (0.42)	<0.001	0.182	1.92 (0.45)	2.01 (0.42)	<0.001	0.189
