# Supplementary material for: Increase in glycemic set point, alongside a decrease in waist circumference, in the non-diabetic population during the Japanese National Intervention Program for metabolic syndrome: A single-center, large-scale, matched-pair analysis
Source: PLoS One. 2022 Aug 10;17(8):e0268450. doi: 10.1371/journal.pone.0268450 (PMC9365144; doi:10.1371/journal.pone.0268450)
Supplement: S2 Table — The results of 1:1 nearest-neighbor matching performed using a caliper of 0.25 were compared to those using a caliper of 0.2. Essentially similar results were obtained. ** Did not achieve the sample size yielded by the power analysis. (PDF) [file pone.0268450.s002.pdf]

**S2 Table**

| | After matching (caliper = 0.2) | | | | After matching (caliper = 0.25) | | | |
|---|---|---|---|---|---|---|---|---|
| | 2007～2008 | 2015～2016 | *P* value | SMD | 2007～2008 | 2015～2016 | *P* value | SMD |
| Women | | | | | | | | |
| *N* | 1024 | 1024 | | | 1076 | 1076 | | |
| Age | 48.37 (10.74) | 48.34 (10.80) | 0.946 | 0.003 | 48.48 (10.86) | 48.45 (10.91) | 0.957 | 0.002 |
| Hb (g/dL) | 13.13 (0.98) | 13.10 (0.97) | 0.632 | 0.021 | 13.12 (0.99) | 13.10 (0.98) | 0.595 | 0.023 |
| RBC ($10^6$/μL) | 4.37 (0.28) | 4.37 (0.29) | 0.672 | 0.019 | 4.37 (0.29) | 4.37 (0.29) | 0.582 | 0.024 |
| Never smoker (%) | 900 (87.9) | 900 (87.9) | | | 939 (87.3) | 939 (87.3) | | |
| Ex-smoker (%) | 87 ( 8.5) | 87 ( 8.5) | 1 | <0.001 | 92 ( 8.6) | 92 ( 8.6) | 1 | <0.001 |
| Current smoker (%) | 37 ( 3.6) | 37 ( 3.6) | | | 45 ( 4.2) | 45 ( 4.2) | | |
| BH (cm) | 158.12 (5.38) | 158.56 (5.35) | 0.065 | 0.082 | 158.02 (5.41) | 158.54 (5.39) | **(0.025) | 0.097 |
| BW (kg) | 53.27 (8.00) | 52.76 (8.07) | 0.151 | 0.063 | 53.13 (7.96) | 52.77 (8.23) | 0.298 | 0.045 |
| BMI | 21.32 (3.14) | 20.98 (3.03) | **(0.014) | 0.108 | 21.29 (3.14) | 20.99 (3.08) | **(0.025) | 0.097 |
| WC (cm) | 77.59 (8.86) | 76.66 (8.78) | 0.018 | 0.105 | 77.54 (8.88) | 76.73 (8.90) | 0.035 | 0.091 |
| SBP (mmHg) | 119.00 (16.03) | 113.81 (15.32) | <0.001 | 0.331 | 118.99 (16.24) | 113.79 (15.43) | <0.001 | 0.328 |
| DBP (mmHg) | 73.58 (10.38) | 69.91 (10.29) | <0.001 | 0.355 | 73.51 (10.40) | 69.91 (10.34) | <0.001 | 0.347 |
| HR (bpm) | 77.20 (11.32) | 63.83 (9.65) | <0.001 | 1.272 | 77.10 (11.28) | 63.96 (9.76) | <0.001 | 1.245 |
| AST (U/L) | 19.94 (6.99) | 19.83 (6.81) | 0.715 | 0.016 | 19.92 (6.87) | 19.85 (6.83) | 0.804 | 0.011 |
| ALT (U/L) | 16.76 (11.06) | 15.87 (10.17) | 0.057 | 0.084 | 16.72 (10.87) | 15.85 (10.17) | 0.056 | 0.083 |
| γ-GTP (U/L) | 22.79 (23.00) | 22.14 (19.57) | 0.486 | 0.031 | 22.32 (20.43) | 22.17 (19.53) | 0.856 | 0.008 |
| Tcho (mg/dL) | 214.19 (36.09) | 209.64 (36.92) | 0.005 | 0.125 | 214.42 (35.93) | 209.25 (36.79) | 0.001 | 0.142 |
| LDL-C (mg/dL) | 120.23 (32.23) | 122.95 (32.65) | 0.058 | 0.084 | 120.58 (32.15) | 122.69 (32.62) | 0.13 | 0.065 |
| HDL-C (mg/dL) | 71.47 (16.45) | 76.27 (16.27) | <0.001 | 0.294 | 71.37 (16.23) | 76.01 (16.21) | <0.001 | 0.286 |
| log TG (log mg/dL) | 4.36 (0.46) | 4.24 (0.45) | <0.001 | 0.248 | 4.35 (0.46) | 4.25 (0.46) | <0.001 | 0.23 |
| FPG (mg/dL) | 87.58 (8.72) | 90.59 (7.94) | <0.001 | 0.36 | 87.67 (8.87) | 90.57 (7.88) | <0.001 | 0.345 |
| HbA1c NGSP (%) | 5.41 (0.36) | 5.51 (0.29) | <0.001 | 0.312 | 5.41 (0.37) | 5.51 (0.28) | <0.001 | 0.308 |
| log F-IRI (log μIU/mL) | 1.55 (0.46) | 1.54 (0.48) | 0.871 | 0.007 | 1.55 (0.46) | 1.54 (0.49) | 0.715 | 0.016 |
| log HOMA-β | 4.30 (0.48) | 4.15 (0.45) | <0.001 | 0.313 | 4.30 (0.47) | 4.15 (0.45) | <0.001 | 0.316 |
| log HOMA-IR | 0.01 (0.51) | 0.04 (0.53) | 0.168 | 0.061 | 0.01 (0.51) | 0.04 (0.53) | 0.242 | 0.05 |
| log adiponectin (log μg/mL) | 2.45 (0.46) | 2.49 (0.44) | **(0.015) | 0.108 | 2.45 (0.46) | 2.49 (0.45) | **(0.036) | 0.091 |
