# Supplementary material for: Increase in glycemic set point, alongside a decrease in waist circumference, in the non-diabetic population during the Japanese National Intervention Program for metabolic syndrome: A single-center, large-scale, matched-pair analysis
Source: PLoS One. 2022 Aug 10;17(8):e0268450. doi: 10.1371/journal.pone.0268450 (PMC9365144; doi:10.1371/journal.pone.0268450)
Supplement: S3 Table — Unlike in Table 1, the data collected at the second and later visits by the same individuals were NOT omitted. This amendment caused an increase in the mean age of the participants from 2007–2008 to 2015–2016 before matching. After matching with respect to age, in addition to smoking status, Hb, and RBC, the risk of comparing the same individuals at different ages was abolished. Similar results to those shown in Table 1 were obtained, except that log F-IRI and log HOMA-IR significantly decreased from 2007–2008 to 2015–2016. The relatively large sample size allowed the BMI data to achieve the sample size yielded by the power analysis. (PDF) [file pone.0268450.s003.pdf]

S3 Table

	Before matching				After matching (caliper = 0.2)			
	2007～2008	2015～2016	*P* value	SMD	2007～2008	2015～2016	*P* value	SMD
Men								
N	8729	9175			5357	5357		
Age	52.95 (11.13)	54.09 (11.36)	<0.001	0.101	53.20 (10.62)	53.26 (10.62)	0.763	0.006
Hb (g/dL)	15.04 (1.03)	14.99 (1.08)	0.002	0.045	15.05 (0.90)	15.02 (0.90)	0.097	0.032
RBC (10^6/μL)	4.70 (0.41)	4.88 (0.42)	<0.001	0.456	4.79 (0.36)	4.80 (0.36)	0.095	0.032
Never smoker (%)	2567 (29.4)	2929 (31.9)			1603 (29.9)	1603 (29.9)		
Ex-smoker (%)	3563 (40.8)	4116 (44.9)	<0.001	0.149	2364 (44.1)	2364 (44.1)	1	<0.001
Current smoker (%)	2599 (29.8)	2130 (23.2)			1390 (25.9)	1390 (25.9)		
BH (cm)	170.04 (6.05)	170.74 (6.06)	<0.001	0.116	170.05 (6.08)	170.96 (6.00)	<0.001	0.15
BW (kg)	68.11 (9.65)	68.85 (10.11)	<0.001	0.075	68.55 (9.63)	68.66 (9.77)	0.564	0.011
BMI	23.52 (2.84)	23.59 (3.01)	0.149	0.022	23.67 (2.81)	23.47 (2.92)	<0.001	0.072
WC (cm)	85.53 (7.85)	84.99 (8.24)	<0.001	0.068	85.93 (7.71)	84.59 (8.05)	<0.001	0.17
SBP (mmHg)	126.60 (14.92)	122.11 (15.04)	<0.001	0.3	127.05 (14.99)	121.75 (14.62)	<0.001	0.358
DBP (mmHg)	79.61 (9.96)	76.55 (10.67)	<0.001	0.297	79.99 (9.98)	76.65 (10.23)	<0.001	0.33
HR (bpm)	72.72 (10.74)	63.61 (9.98)	<0.001	0.879	73.12 (10.79)	63.66 (9.85)	<0.001	0.915
AST (U/L)	23.92 (10.44)	23.36 (9.18)	<0.001	0.057	23.46 (9.52)	23.52 (9.63)	0.765	0.006
ALT (U/L)	25.76 (17.91)	24.27 (16.30)	<0.001	0.087	25.88 (17.67)	24.07 (16.48)	<0.001	0.106
γ-GTP (U/L)	53.65 (63.99)	48.02 (54.82)	<0.001	0.095	50.40 (49.84)	50.38 (55.98)	0.989	<0.001
Tcho (mg/dL)	211.98 (32.04)	205.08 (31.67)	<0.001	0.217	213.74 (32.31)	204.43 (30.97)	<0.001	0.294
LDL-C (mg/dL)	127.41 (30.39)	126.23 (28.84)	0.007	0.04	129.57 (30.30)	125.43 (28.34)	<0.001	0.141
HDL-C (mg/dL)	58.93 (13.85)	61.16 (15.33)	<0.001	0.153	57.89 (13.37)	62.12 (15.60)	<0.001	0.291
log TG (log mg/dL)	4.76 (0.53)	4.67 (0.53)	<0.001	0.182	4.78 (0.52)	4.66 (0.53)	<0.001	0.23
FPG (mg/dL)	94.51 (9.70)	96.18 (8.90)	<0.001	0.179	94.70 (9.63)	96.01 (8.95)	<0.001	0.142
HbA1c NGSP (%)	5.54 (0.40)	5.60 (0.30)	<0.001	0.156	5.56 (0.39)	5.56 (0.30)	0.848	0.004
log F-IRI (log μIU/mL)	1.67 (0.53)	1.68 (0.55)	0.141	0.022	1.70 (0.52)	1.65 (0.55)	<0.001	0.102
log HOMA-β	4.16 (0.54)	4.10 (0.52)	<0.001	0.103	4.18 (0.53)	4.07 (0.52)	<0.001	0.206
log HOMA-IR	0.21 (0.57)	0.24 (0.59)	<0.001	0.052	0.24 (0.56)	0.20 (0.59)	<0.001	0.07
log adiponectin (log μg/mL)	1.97 (0.46)	2.03 (0.44)	<0.001	0.133	1.96 (0.45)	2.03 (0.42)	<0.001	0.17
