# Supplementary material for: Increase in glycemic set point, alongside a decrease in waist circumference, in the non-diabetic population during the Japanese National Intervention Program for metabolic syndrome: A single-center, large-scale, matched-pair analysis
Source: PLoS One. 2022 Aug 10;17(8):e0268450. doi: 10.1371/journal.pone.0268450 (PMC9365144; doi:10.1371/journal.pone.0268450)
Supplement: S4 Table — Unlike in Table 2, the data collected at the second and later visits by the same individuals were NOT omitted. This amendment caused an increase in the mean age from 2007–2008 to 2015–2016 before matching. After matching with respect to age, in addition to smoking status, Hb, and RBC, the risk of comparing the same individuals at different ages was abolished. Similar results to those shown in Table 2 were obtained, except that BH significantly increased and γ-GTP significantly decreased from 2007–2008 to 2015–2016. The relatively large sample size permitted BW, BMI, ALT, and log adiponectin to achieve the sample size yielded by the power analysis. ** Did not achieve the sample size yielded by the power analysis. (PDF) [file pone.0268450.s004.pdf]

S4 Table

	Before matching				After matching (caliper = 0.2)			
	2007～2008	2015～2016	*P* value	SMD	2007～2008	2015～2016	*P* value	SMD
Women								
N	5733	6390			3246	3246		
Age	50.52 (11.56)	52.31 (11.45)	<0.001	0.156	51.31 (10.73)	51.37 (10.71)	0.831	0.005
Hb (g/dL)	13.16 (1.15)	13.15 (1.12)	0.703	0.007	13.16 (1.02)	13.13 (1.00)	0.196	0.032
RBC (10^6/μL)	4.24 (0.33)	4.44 (0.35)	<0.001	0.603	4.32 (0.30)	4.33 (0.30)	0.247	0.029
Never smoker (%)	4846 (84.5)	5244 (82.1)			2866 (88.3)	2866 (88.3)		
Ex-smoker (%)	449 (7.8)	774 (12.1)	<0.001	0.155	242 (7.5)	242 (7.5)	1	<0.001
Current smoker (%)	438 (7.6)	372 (5.8)			138 (4.3)	138 (4.3)		
BH (cm)	157.71 (5.53)	158.02 (5.62)	0.002	0.056	157.62 (5.44)	158.23 (5.51)	<0.001	0.111
BW (kg)	52.46 (7.74)	52.85 (8.30)	0.008	0.049	52.99 (7.97)	52.28 (7.83)	<0.001	0.09
BMI	21.10 (3.01)	21.16 (3.16)	0.243	0.021	21.34 (3.09)	20.88 (2.96)	<0.001	0.15
WC (cm)	77.49 (8.86)	77.55 (8.90)	0.692	0.007	78.14 (9.01)	76.67 (8.63)	<0.001	0.167
SBP (mmHg)	119.77 (16.72)	116.35 (16.68)	<0.001	0.205	120.96 (16.88)	114.74 (16.06)	<0.001	0.377
DBP (mmHg)	74.07 (10.66)	71.22 (10.87)	<0.001	0.265	74.87 (10.81)	70.83 (10.63)	<0.001	0.377
HR (bpm)	75.75 (11.16)	64.06 (9.62)	<0.001	1.123	76.40 (11.30)	63.78 (9.52)	<0.001	1.208
AST (U/L)	20.64 (8.30)	20.38 (7.02)	0.056	0.035	20.41 (6.22)	20.22 (6.19)	0.221	0.03
ALT (U/L)	17.11 (10.85)	16.34 (10.22)	<0.001	0.073	17.24 (10.21)	15.97 (8.99)	<0.001	0.131
γ-GTP (U/L)	23.98 (25.03)	23.30 (24.64)	0.134	0.027	24.20 (25.24)	22.91 (21.73)	0.027	0.055
Tcho (mg/dL)	216.66 (35.86)	212.72 (35.22)	<0.001	0.111	219.64 (36.08)	210.62 (34.93)	<0.001	0.254
LDL-C (mg/dL)	120.17 (31.78)	125.01 (31.19)	<0.001	0.154	123.36 (32.25)	123.00 (30.74)	0.645	0.011
HDL-C (mg/dL)	72.34 (16.46)	76.38 (17.35)	<0.001	0.239	71.82 (16.34)	77.55 (17.41)	<0.001	0.34
log TG (log mg/dL)	4.36 (0.47)	4.30 (0.45)	<0.001	0.143	4.39 (0.47)	4.26 (0.44)	<0.001	0.293
FPG (mg/dL)	88.28 (8.88)	90.90 (8.12)	<0.001	0.308	88.65 (8.90)	90.48 (8.06)	<0.001	0.215
HbA1c NGSP (%)	5.39 (0.38)	5.57 (0.29)	<0.001	0.541	5.43 (0.36)	5.54 (0.28)	<0.001	0.328
log F-IRI (log μIU/mL)	1.51 (0.49)	1.55 (0.50)	<0.001	0.08	1.53 (0.48)	1.50 (0.49)	**(0.035)	0.052
log HOMA-β	4.23 (0.50)	4.15 (0.47)	<0.001	0.17	4.24 (0.48)	4.12 (0.46)	<0.001	0.243
log HOMA-IR	-0.02 (0.53)	0.05 (0.55)	<0.001	0.13	0.01 (0.53)	0.00 (0.53)	0.757	0.008
log adiponectin (log μg/mL)	2.47 (0.46)	2.50 (0.46)	0.002	0.058	2.45 (0.47)	2.52 (0.45)	<0.001	0.137
